# Two Family Gradients in Children’s Physical Activity and Fitness: Socioeconomic Position, Family Structure, and Where They Diverge

**DOI:** 10.3390/healthcare14193189

**Published:** 2026-09-27

**Authors:** Yanfeng Zhang, Xiang Pan, Yibo Gao, Haixia Hu

**Affiliations:** 1 China Institute of Sport Science, No. 11 Tiyuguan Road, Dongcheng District, Beijing 100061, China; 2 Graduate School of Health and Sports Science, Juntendo University, Inzai 270-1695, Chiba, Japan; panxiang9804@163.com (X.P.); gaoyibo1019@163.com (Y.G.); 3 Weifang Aiyoudong Children and Youth Sports Health Research Institute, Weifang 261061, China

**Keywords:** children, physical activity, physical fitness, handgrip strength, socioeconomic position, family structure, sibship size, physical education, health inequality, China

## Abstract

**Highlights:**

**What are the main findings?**

- Family socioeconomic position, indexed by parental education, was associated with children’s reported participation in exercise organised by families; no gradient was observed in the physical education class that every child attends.
- Only children showed no clear difference in reported activity compared with children with siblings, yet they had lower measured fitness, and the gap remained after adjustment for reported activity.

**What are the implications of the main findings?**

- Reported activity statistically accounted for little of the family gap in measured fitness; so, surveillance of reported minutes alone would not identify the source of that gap.
- Examining reported activity by setting and measured fitness by dimension shows where each family gradient appears: in which activity settings and on which fitness dimensions.

**Abstract:**

Background/Objectives: Family circumstances are related to children’s physical activity and fitness, yet the two are rarely examined together in the same children. We examined how socioeconomic position and family structure (only-child status) relate to domain-specific reported activity and measured fitness. Socioeconomic position was the mean of maternal and paternal educational attainment, with higher values indicating higher attainment. Methods: We designed and conducted a cross-sectional survey of 8228 children in grades 1 to 9 from 27 schools. The questionnaire covered the preceding six months in three domains: physical education class, in-school extracurricular activity and out-of-school exercise. Weekly frequency, duration and intensity were reported for each and summed to weekly moderate-to-vigorous activity (MVPA). Fitness comprised 10 device-measured indicators and, in primary grades, a 5-item motor battery. Linear and logistic models used school fixed effects and school-clustered standard errors, with Benjamini–Hochberg correction. Results: Median reported weekly MVPA was 300 min; 26.3% of the 7533 children with computable MVPA met the 60 min-per-day guideline. Socioeconomic position was associated with reported MVPA (0.077 SD, 95% CI 0.032 to 0.122) and with meeting the guideline (odds ratio 1.21, 95% CI 1.07 to 1.37). The association appeared in out-of-school exercise but not in physical education class, a contrast of 0.064 SD (95% CI 0.024 to 0.104). Socioeconomic position was also associated with lower handgrip strength (−0.044 SD, 95% CI −0.064 to −0.024) and with more sit-ups in primary grades. Only children showed no clear difference in reported activity in any domain, yet they had lower composite fitness (−0.102 SD, 95% CI −0.142 to −0.061), which remained after adjustment for reported MVPA. Conclusions: The socioeconomic gradient in reported activity was concentrated outside the physical education class; family structure was associated with measured fitness without a matching difference in reported activity.

## 1. Introduction

Childhood physical activity and physical fitness are each socially patterned, and both are associated with health later in life [1,2,3]. National surveillance in China has documented persistent social and regional differences in both physical activity and fitness [4,5]. Because these differences emerge early and are difficult to reverse, identifying which family circumstances correspond to which outcome is a prerequisite for directing support toward the children who need it [6].

Children accumulate physical activity in settings that differ in who provides the opportunity. Physical education classes are provided by the school on a common timetable. Extracurricular activity within school combines school provision with family initiative, and exercise outside school depends more heavily on what a household is able to organise and sustain [7,8,9]. Because a single weekly total obscures these differences between settings, family gradients of the same magnitude can occur in settings that policy can influence to very different degrees [10,11].

Physical fitness also comprises several dimensions. Strength, cardiorespiratory capacity, flexibility and body composition develop on different timescales and respond to different demands, so that a child who scores well on one of them may not score well on another [12,13,14]. National reporting systems nonetheless summarise these dimensions in a single score, which provides one number for surveillance even when the associations across dimensions may differ in direction [15]. Few studies have formally tested whether family circumstances relate to only some of these dimensions, or whether the same family variable relates to different dimensions in opposite directions, in a sample large enough for direct comparison between dimensions.

Family circumstances themselves include several distinct characteristics. Socioeconomic position describes the resources a household can mobilise. Family structure describes the household composition, and the presence of siblings has been linked to children’s activity and motor development through everyday companionship, without additional household spending [16,17,18]. The two are seldom examined side by side in the same children, and their gradients in activity and in fitness may not coincide. Studies of Chinese children have linked parental education, household income and household composition to fitness-related outcomes in a national sample and in samples of rural left-behind minors and preschool children [19,20,21].

These gaps in knowledge have practical as well as descriptive importance. If a family gradient in activity arises mainly in the exercise that households organise, then changes to the school timetable will not close it. If a family difference in fitness appears without a matching difference in reported activity, then the reported volume cannot fully account for that difference. Testing both possibilities requires the reported activity to be assessed by setting and fitness by dimension, with both measured in the same children.

Three questions were therefore examined in 8228 schoolchildren: whether socioeconomic position and family structure are associated with reported physical activity and in which domain any difference appears; whether they are associated with measured fitness and on which dimensions; and how much of the family differences in fitness is statistically accounted for by the reported activity.

## 2. Materials and Methods

### 2.1. Design and Participants

The study was conducted in accordance with the Declaration of Helsinki. The protocol was approved by the Ethics Committee of the China Institute of Sport Science (protocol code CISSLA20230110, approved on 10 January 2023), and written informed consent was obtained from the parent or guardian of every participant. The data come from a school-based survey that we designed and conducted in Weifang, Shandong Province, in 2023. The sampling frame was the register of full-time primary and junior-high schools in five administrative districts of the city: two in the urban core and three in suburban or peri-urban areas. The schools were stratified by district type and by educational stage, and 27 schools were selected. Within each selected school, whole classes were enrolled across grades 1 to 9, covering three educational stages: lower primary (grades 1 to 3), upper primary (grades 4 to 6) and junior high (grades 7 to 9). Children were eligible if they were enrolled in grades 1 to 9 at a sampled school and took part in the fitness testing, and no further exclusion criteria were applied at enrolment. In the lower primary grades, the questionnaire was completed with a parent, who read each item to the child and recorded the child’s answer. Children in grades 4 to 9 completed the activity items themselves in class under the supervision of trained investigators. In all grades, the items on family circumstances were completed by a parent or with parental assistance, and fitness testing was carried out at the school by staff trained in the national testing protocol.

Of the 10,478 children in grades 1 to 9 enrolled at the 27 schools, 9340 had a fitness score, and 8412 had a fitness score together with a socioeconomic measure, a body-mass index and family-structure data. Of these, 8228 had complete data on all core analysis variables and formed the analytic sample (4175 boys and 4053 girls; mean age 10.59 years, SD 2.56; 27 schools; 27.9 percent were only children). The 184 excluded records lacked a usable score for the body-mass-index item (130) or age data (57, with 3 lacking both). Compared with those retained, the excluded children had lower parental education and, where measurable, lower fitness scores (Supplementary Table S5). Figure 1 shows the participant selection, and each record corresponds to one child.

### 2.2. Physical Activity

Physical activity was reported for three separate domains over the preceding six months: the physical education class, in-school extracurricular activity (excluding morning and between-class exercises), and exercise outside school (including exercise on the school grounds after class). Each domain was assessed with three items: the number of days per week, the usual duration of a session, and the physical sensations usually experienced during the activity. Days were coded from 0 to 5, and duration categories were assigned their midpoints of 15, 45, 90 and 150 min. Intensity was recorded on three levels: little change in breathing and heart rate; faster breathing and heart rate with light sweating; and rapid breathing with a markedly faster heart rate and heavier sweating. The upper two levels were classified as moderate-to-vigorous. Children who reported zero days in a domain skipped the duration and intensity items for that domain, and their weekly minutes for it were recorded as zero; missing values arose only from unanswered items. Otherwise, weekly minutes for a domain were calculated by multiplying the number of days by the midpoint of the session-duration category, and the weekly MVPA was calculated by summing minutes across the domains reported at moderate or vigorous intensity. Meeting the guideline was defined as 420 min of MVPA per week, which is equivalent to the World Health Organization recommendation of an average of 60 min per day [22]. Weekly totals were winsorised at the 99.5th percentile to limit the influence of implausibly high products of the category midpoints; estimates without winsorisation were essentially identical (Supplementary Table S6). Questionnaire items and administration followed the survey’s fieldwork manual, which specified the use of the same instrument in every grade from 1 to 9, a printed six-month recall window, and supervised completion as described in Section 2.1. The instrument has not been standardised against a criterion measure such as accelerometry or direct observation. Activity variables are therefore treated throughout as reported activity, the estimates that depend on activity volume await replication with device-based measurement, and calibration of the instrument against a criterion measure is a priority for further work. Within this sample, the instrument reproduced the expected patterns of children’s reported activity. Boys reported 15.1 min more MVPA per week than girls of the same age within the same school (95% CI 4.2 to 26.0). The reported MVPA and guideline adherence were lowest in junior high, at 293.9 versus 334.2 min per week and 17.8% versus 30.5% in lower primary (Supplementary Table S4). These are construct-related patterns and are not a substitute for criterion validation.

### 2.3. Physical Fitness

Fitness was measured by trained staff following the 2014 National Student Physical Health Standard testing protocol [23]. Analyses across grades 1 to 9 included ten device-measured indicators: handgrip strength, handgrip strength per kilogram of body mass, vital capacity, vital capacity per kilogram of body mass, systolic blood pressure, diastolic blood pressure, waist-to-hip ratio, waist-to-height ratio, body fat percentage and body-mass index. Measured waist and hip circumferences were incorporated into the analyses through the two ratios. Handgrip strength was measured with an electronic dynamometer (GMCS-WCS3; Xindong Huateng, Beijing, China). Body fat percentage was measured by bioelectrical impedance using the instrument deployed for national fitness surveillance (Xindong Huateng, Beijing, China), and the same equipment model was used at every school. A 5-item motor battery was administered in the primary grades: standing long jump, 50 m sprint, 50 m by 8 shuttle run, sit-and-reach and one-minute sit-ups. Sprint and shuttle times were converted to speeds so that higher values indicate better performance. A composite fitness score was retained for comparability; it was computed from the national standard with the body-mass-index item removed and the remaining weights renormalised.

All fitness and activity outcomes were standardised within cells defined by sex and integer age, so that a coefficient expresses a difference in standard deviations relative to same-sex same-age peers.

### 2.4. Family Exposures and Covariates

The socioeconomic position was represented by parental education, the primary socioeconomic indicator in this study. The measure was calculated as the mean of maternal and paternal educational attainment, each recorded on a four-level scale (junior high school or below; senior or vocational high school; college or associate degree; postgraduate degree), and the mean was entered as a continuous variable, with higher values indicating higher attainment. Parental education captures one dimension of socioeconomic position, and statements about socioeconomic position in this paper refer to this education-based measure. Maternal and paternal education were also examined separately. Maternal and paternal occupations were recorded in nine categories, recoded to a four-level prestige score and examined separately as well (Supplementary Table S7). Household income was collected only in the junior-high subsample and was included in a sensitivity analysis. No other indicators of household material circumstances were collected. Family structure was recorded as only-child status and, in ordinal form, as the number of children in the household (one child 27.9%, two children 60.8%, three or more 11.3%). All models were adjusted for age, school fixed effects, and the other family exposure, so that each estimate is conditional on the other.

### 2.5. Statistical Analysis

Continuous outcomes were analysed with linear regression, and the binary indicator of meeting the guideline, defined by dichotomising weekly MVPA at 420 min, was analysed with logistic regression; both used the same covariates, school fixed effects and school-clustered robust standard errors [24]. Outcome distributions were inspected before modelling. Because several activity and fitness variables are skewed, descriptive statistics report medians and interquartile ranges alongside means, and inference rests on cluster-robust standard errors, which do not require normally distributed or homoscedastic residuals. Diagnostics for the principal models, namely variance-inflation factors for the exposures (all at or below 1.2), residual skewness and kurtosis, and Breusch–Pagan tests, are reported in Supplementary Table S9. Statistical significance was assessed using a two-sided significance level of 0.05, with 95% confidence intervals reported throughout. Adiposity outcomes were not adjusted for body-mass index. Sex-stratified estimates and multiplicative exposure-by-sex interaction terms were computed for the complete set of outcomes; given the number of interactions examined, these analyses are exploratory, and individual interaction terms are interpreted as such. Multiple testing was addressed using the Benjamini–Hochberg procedure to control the false discovery rate at 0.05 within each prespecified family of tests, separately for each exposure. The seven activity outcomes were total weekly activity minutes, weekly MVPA, the three domain-specific totals, walking time to the child’s usual exercise venue and use of community sports facilities; the ten device-measured indicators and the five motor-battery items are those listed in Section 2.3. The composite fitness score was a single prespecified outcome, and its *p* value is reported without correction [25]. Differences in gradients across settings or fitness dimensions were tested using stacked models restricted to children with complete data on every element, with dimension-specific covariates and standard errors clustered by child. For each dimension d and the reference dimension r, the contrast and its variance were obtained from the linear combination of the two exposure slopes. Composite construct axes were formed by averaging standardised outcomes, and the associations with these axes were re-estimated after omitting each element in turn; the indicators included in each axis are listed in Supplementary Table S8. The share of each family difference statistically attributable to the weekly reported MVPA was estimated using a cross-sectional product-of-coefficients decomposition of the association, after residualising the outcome, the exposure and MVPA with respect to age and school. Percentile confidence intervals were obtained from 1000 school-cluster bootstrap resamples. Because the design is cross-sectional, this decomposition partitions associations and implies no temporal or causal ordering, a caveat that applies to every indirect estimate reported below. Because the motor battery was administered in the primary grades, the stacked comparison of fitness dimensions was restricted to children with complete data on all eight compared dimensions, and its scope is reported alongside the estimates. Additional analyses examined differences by school stage, the junior-high subsample with reported household income, socioeconomic position defined by the highest level of household educational attainment, and performance outcomes with and without adjustment for body-mass index. Further analyses compared included and excluded records, used occupation-based indicators, omitted the winsorisation, restricted the sample to children born after the 2013 relaxation of the one-child policy, and provided sex-stratified estimates with full confidence intervals (Supplementary Tables S2, S3, S5–S7, S10 and S11). Analyses were performed using R 4.4.2, and the study is reported in accordance with STROBE (Supplementary Table S1).

## 3. Results

### 3.1. Activity and Fitness Profile

Children reported a median of 300 min of MVPA per week (interquartile range 165 to 450), and 26.3% of the 7533 children with computable MVPA reached 420 min. The mean reported activity time was 132.1 min per week (SD 59.8) in the physical education class, 129.2 (SD 99.6) in in-school extracurricular activity and 119.5 (SD 112.1) in exercise outside school. The mean handgrip strength was 16.06 kg (SD 8.35), the mean body fat percentage 15.77% (SD 7.55) and the mean diastolic blood pressure 70.02 mm Hg (SD 8.82). The measurement battery and its coverage are shown in Table 1 and the characteristics of the sample in Table 2. Motor-battery items were available for 4540 to 5796 primary-grade children, and per-outcome sample sizes are given in Table 1.

### 3.2. Family Correlates of Reported Physical Activity, by Domain

The socioeconomic position was associated with the weekly MVPA (0.077 SD, 95% CI 0.032 to 0.122, FDR-adjusted *p* = 0.006, *n* = 7532) and with the total reported activity (0.069 SD, 95% CI 0.019 to 0.119, FDR-adjusted *p* = 0.023), and each unit of parental education was associated with higher odds of meeting the guideline (odds ratio 1.21, 95% CI 1.07 to 1.37, *p* = 0.003). Across domains, the estimate was 0.018 SD (95% CI −0.021 to 0.058, *p* = 0.36) for the physical education class, 0.048 SD (95% CI 0.010 to 0.086, FDR-adjusted *p* = 0.031) for in-school extracurricular activity and 0.075 SD (95% CI 0.013 to 0.137, FDR-adjusted *p* = 0.031) for exercise outside school. Relative to the physical education class, the contrast was 0.064 SD (95% CI 0.024 to 0.104, *p* = 0.002) for out-of-school exercise and 0.031 SD (95% CI −0.003 to 0.064, *p* = 0.07) for in-school extracurricular activity. The share of MVPA contributed by the physical education class was 34.3%, 35.3% and 35.5% in the low, middle and high socioeconomic groups, respectively.

Compared with children who had siblings, only children showed no clear difference in reported activity in the physical education class (−0.007 SD, 95% CI −0.046 to 0.032), in in-school extracurricular activity (−0.010 SD, 95% CI −0.062 to 0.042) or in exercise outside school (−0.005 SD, 95% CI −0.048 to 0.038). The domain contrasts relative to the physical education class were 0.003 SD (95% CI −0.055 to 0.061) and 0.001 SD (95% CI −0.049 to 0.052). The only-child estimate for weekly MVPA was −0.040 SD (95% CI −0.076 to −0.003, FDR-adjusted *p* = 0.22), and the odds ratio for meeting the guideline was 0.98 (95% CI 0.89 to 1.09, *p* = 0.72). Because the absence of clear evidence of a difference is not evidence of equivalence, the confidence intervals indicate how large an undetected difference could plausibly be. Figure 2 shows these estimates.

### 3.3. Family Correlates of Fitness, by Dimension

The socioeconomic position was associated with lower handgrip strength (−0.044 SD, 95% CI −0.064 to −0.024, FDR-adjusted *p* < 0.001, *n* = 8150), with lower handgrip per kilogram of body mass and lower diastolic blood pressure (−0.053 SD, 95% CI −0.091 to −0.015, FDR-adjusted *p* = 0.031) and with more sit-ups in the primary-grade subsample (0.054 SD, 95% CI 0.021 to 0.087, FDR-adjusted *p* = 0.007, *n* = 4540). In an exploratory sex-stratified comparison, the sit-up gradient was 0.086 SD in girls and −0.003 SD in boys (interaction *p* < 0.001). In raw units, the socioeconomic gradients in handgrip strength and in sit-ups correspond to −0.20 kg (95% CI −0.29 to −0.11) and 0.45 repetitions (95% CI 0.17 to 0.73), respectively, per unit of parental education. Estimates for the four adiposity indicators ranged from −0.029 SD to 0.005 SD, with all intervals including zero, and the estimate for the composite score was 0.030 SD (95% CI −0.0003 to 0.061, *p* = 0.052). Across the observed range of parental education, the adjusted means ranged from 0 to −0.118 SD for handgrip strength and from 0 to 0.107 SD for sit-ups. Sex-stratified estimates with full confidence intervals are given in Supplementary Table S11.

Only children had lower composite fitness scores (−0.102 SD, 95% CI −0.142 to −0.061, FDR-adjusted *p* < 0.001; in raw units 1.28 points lower on the renormalised 0–100 national score, 95% CI 0.77 to 1.79), lower handgrip strength (−0.071 SD, 95% CI −0.119 to −0.024, FDR-adjusted *p* = 0.034; 0.29 kg, 95% CI 0.05 to 0.52) and lower 50 m sprint speed (−0.091 SD, 95% CI −0.150 to −0.033, FDR-adjusted *p* = 0.011). They also performed less well on shuttle speed (FDR-adjusted *p* = 0.043) and sit-and-reach (FDR-adjusted *p* = 0.032); the confidence intervals for standing long jump and sit-ups only just included zero. Estimates for the four adiposity indicators ranged from 0.008 SD to 0.029 SD, with all intervals including zero. Table 3 and Figure 3 present the complete set of estimates.

### 3.4. Whether Gradients Differ Across Dimensions

Among primary-school children with complete data on all eight dimensions compared (*n* = 4256; 34,048 observations), the socioeconomic slope differed from that for handgrip strength by 0.090 SD for sit-ups (95% CI 0.031 to 0.149, *p* = 0.003), 0.072 SD for standing long jump (*p* = 0.017), 0.067 SD for shuttle speed (*p* = 0.030) and 0.064 SD for sprint speed (*p* = 0.032). The corresponding contrasts were 0.057 SD for sit-and-reach (*p* = 0.07), 0.023 SD for waist-to-height ratio and 0.034 SD for body fat percentage. For only-child status, the seven corresponding contrasts ranged from −0.044 SD to 0.070 SD, with *p* values from 0.11 to 0.63. On the motor-performance axis the only-child estimate was −0.087 SD (95% CI −0.140 to −0.034, *p* = 0.001), and omitting each element in turn gave estimates from −0.088 SD to −0.083 SD, all with *p* values of 0.004 or below. The indicators entering each construct axis are listed in Supplementary Table S8. Figure 4 shows the contrasts and the leave-one-out estimates.

### 3.5. Whether Reported Activity Statistically Accounts for the Family Differences in Fitness

The association between family circumstances and reported MVPA was 0.077 SD (95% CI 0.032 to 0.122) for socioeconomic position, equivalent to 16.8 min per week per unit of parental education (95% CI 7.1 to 26.5), and −0.040 SD (95% CI −0.076 to −0.003) for only-child status. Association estimates between reported MVPA and fitness ranged from 0.011 SD to 0.048 SD across the nine outcomes, and only the association with standing long jump met the false-discovery-rate threshold (0.048 SD, 95% CI 0.031 to 0.065). In the cross-sectional decomposition, which partitions associations and implies no temporal or causal ordering, the components attributable to reported MVPA ranged from −0.001 SD to 0.006 SD; the interval excluded zero for standing long jump (0.006 SD, 95% CI 0.002 to 0.009) and for the composite score (0.003 SD, 95% CI 0.001 to 0.007).

After adjustment for weekly reported MVPA, the socioeconomic estimate for handgrip strength was −0.052 SD (95% CI −0.072 to −0.031), compared with −0.044 SD before adjustment. The only-child estimate for the composite score was −0.085 SD (95% CI −0.126 to −0.045, *p* < 0.001), compared with −0.102 SD before adjustment. In the junior-high subsample with reported household income (*n* = 2197), the socioeconomic estimate for handgrip strength was −0.053 SD (95% CI −0.104 to −0.002) before income adjustment and −0.043 SD after adjustment. Defining socioeconomic position by the highest level of household educational attainment and adjusting the performance outcomes for body-mass index did not change the direction of any estimate, and the magnitudes were similar (Supplementary Tables S2 and S3). Neither occupation-based indicator, maternal or paternal occupational prestige, showed an association with reported activity or with fitness that met the false-discovery-rate threshold (Supplementary Table S7). Restricting the sample to children born after the 2013 relaxation of the one-child policy left the only-child estimates essentially unchanged (composite score −0.100 SD, 95% CI −0.150 to −0.051; Supplementary Table S10). Figure 5 shows the two component associations and the adjusted comparisons.

## 4. Discussion

Socioeconomic position, indexed by parental education, and family structure were associated with different outcomes in the same children. The socioeconomic position was associated with the reported weekly MVPA and with the odds of meeting the guideline. This association appeared in exercise outside school and in extracurricular activity within school. The physical education class showed no gradient, and the contrast between out-of-school exercise and the class was 0.064 SD. Socioeconomic estimates for fitness differed in sign across dimensions: they were negative for handgrip strength and diastolic blood pressure and positive for sit-ups. Estimates for all four adiposity indicators had intervals including zero, and among primary-school children each motor item had a slope that differed from the slope for handgrip strength. Family structure was associated with a different pattern in the same children: only children showed no clear difference in reported activity in any domain, yet had lower composite fitness scores, handgrip strength, sprint speed, shuttle speed and sit-and-reach performance. Because the seven dimension contrasts were indistinguishable from one another, the difference was of a common magnitude across the battery, with no evidence of concentration in any single test. Omitting any single element of the motor battery left the only-child estimate between −0.088 SD and −0.083 SD. Adjustment for weekly reported MVPA changed the socioeconomic estimate for handgrip strength from −0.044 SD to −0.052 SD and the only-child estimate for the composite score from −0.102 SD to −0.085 SD.

The setting in which a socioeconomic gradient in reported activity appears depends on who provides the activity. The physical education class is timetabled for every child in a school, and within schools it showed no socioeconomic gradient. A socioeconomic gradient was observed in exercise arranged outside school. Analyses by activity domain in other settings report the same pattern, with participation in organised and out-of-school activity more closely associated with household resources than participation in curricular activity [26,27,28,29,30]. School fixed effects account for differences between schools; so, the estimate compares children attending the same school and describes a difference at the household level, conditional on what a school offers. The amount of activity reported in a setting matters alongside its socioeconomic gradient: children reported a mean of 132.1 min per week in the physical education class, and its share of MVPA varied by only 1.2 percentage points across the three socioeconomic groups. An analysis has argued that activity during curricular provision is least responsive to household circumstances because attendance is not optional [31]; in the present study, the contrast compares activity settings assessed in the same children over the same period. The World Health Organization recommends an average of 60 min of moderate-to-vigorous activity per day for school-aged children [22]. In this sample, 26.3% of children with computable MVPA reached the equivalent weekly threshold; so, the observed gradient occurred in a population in which most children reported less than the recommended volume.

The socioeconomic gradient in fitness was dimension-specific in a way that the family-structure difference was not. The handgrip estimate had the opposite sign to the estimates for the trained-performance items, and the estimate for the composite score, which averages over both, was close to zero. Handgrip strength was examined as a dimension in its own right because it indexes overall muscular strength in children and relates to cardiometabolic risk [32]. Handgrip strength in children has been reported to relate to habitual loading and everyday physical work as much as to organised exercise [32,33,34,35], which is consistent with a dimension that reflects loading outside organised activity. That the four adiposity indicators showed no clear socioeconomic gradient also differs from the pattern reported in higher-income settings, where socioeconomic position is inversely associated with childhood adiposity [36,37,38,39], and is closer to reports from settings in which that gradient is weak or absent [40]. Direct comparisons within the same children showed that the slopes for sit-ups, standing long jump, shuttle speed and sprint speed each differed from the slope for handgrip strength; so, the pattern is not explained by differences in sample size or precision between tests. Applying the same comparison to family structure yielded seven contrasts that were indistinguishable from one another, consistent with a difference of common magnitude across the battery.

Test engagement, everyday physical loading and reporting should be considered alongside household resources as explanations for the handgrip finding. Test engagement could contribute because sit-ups, sit-and-reach and the shuttle run depend on sustained effort and familiarity with the protocol, but the brief maximal squeeze used to measure handgrip strength is comparatively insensitive to how willingly a child completes a battery; a socioeconomic gradient in engagement would therefore raise the trained items relative to the grip item [41,42]. Differences in everyday physical loading could also contribute, since children in lower-resource households may do more carrying, lifting and physically demanding household work, and grip strength responds to such loading [43]. Differences in reporting require particular consideration here. Because activity was reported by families for the youngest children and by the children themselves in grades 4 to 9, higher-resource households may report organised activity more completely, which would inflate the domain-specific gradient without a matching difference in physical activity [44,45]. The socioeconomic gradient appeared in the domain most dependent on recall and on family report and was not evident in the timetabled class. No clear gradient appeared in the adiposity indicators, which might have been expected to vary with a true difference in activity-related energy expenditure. Within this design, explanations based on reporting and on household resources cannot be separated; so, the domain-specific gradient is interpreted throughout as a gradient in reported activity.

In the cross-sectional decomposition, which precludes temporal or causal inference, reported activity statistically accounted for very little of the family differences in fitness. Examining the two component associations separately explains this result. The socioeconomic position was associated with the reported MVPA, but the associations between the reported MVPA and measured fitness were small, ranging from 0.011 SD to 0.048 SD per standard deviation of MVPA, and only the association with the standing long jump met the false-discovery-rate threshold. Statistical indirect components were correspondingly small, and the estimates for handgrip strength and the composite score changed little after MVPA was included in the models. Reviews of the association between questionnaire-based activity and measured fitness in youth report similarly modest correlations [46,47,48], consistent with the weak association observed here. The discrepancy is more pronounced for family structure: only children showed no clear difference in reported activity yet performed less well on the fitness tests; so, their lower fitness was not accompanied by any clear difference in reported activity volume. The reported volume may be an imprecise proxy for the loading that develops physical capacity, in which case, the measurement error would attenuate the association between the reported activity and measured fitness [49]. Alternatively, this association may be weak at this age because improvements in measured strength and motor performance may depend on the type and intensity of loading, which accumulated minutes capture poorly; device-based studies report a similar distinction when total volume and time spent in vigorous activity are examined separately [50,51,52,53]. The comparison before and after adjustment (Figure 5D) accounts only for the total weekly MVPA minutes, and adjustment for aggregate volume alone does not preclude mediation by qualitative or modality-specific characteristics of movement that the questionnaire did not capture, such as high-impact bone loading, neuromuscular loading or unstructured peer play. Under either interpretation, reported minutes alone will provide an incomplete description of family differences in children’s fitness. These decompositions describe how associations are partitioned in this data set; they do not estimate what any intervention would achieve.

Several exploratory sex interactions indicated comparatively large differences: the socioeconomic gradient in sit-ups was concentrated in girls, with no clear gradient in boys (0.086 versus −0.003 SD, interaction *p* < 0.001); the gradient in in-school extracurricular activity was stronger in boys (interaction *p* = 0.014); and the only-child difference in sit-ups was larger in boys (interaction *p* = 0.004). Because these interactions were examined across the complete set of outcomes, individual interaction terms are exploratory and are not interpreted as standalone findings. The socioeconomic pattern observed predominantly among girls is nonetheless consistent with the sex-differentiated allocation of household resources reported in other settings [17], and it warrants examination using data collected for that purpose.

Only-child status is also not randomly distributed. It was more common among boys than among girls (32.6 versus 22.9 percent), which is consistent with fertility behaviour during the one-child-policy era [54], and one-child and multi-child families may differ in parental age, residence and patterns of family investment. Restricting the analysis to children born after the policy was relaxed in 2013 left the only-child estimates essentially unchanged (Supplementary Table S10); this restriction addresses cohort composition, although it does not address family-level selection within cohorts.

The cross-sectional design does not establish the temporal ordering of the variables. The sample comes from a single city, and the distributions of socioeconomic position and family structure differ elsewhere in China. Physical activity was assessed by questionnaire without device measurement. The six-month recall window allows for recall error, duration was represented by category midpoints, and intensity was recorded once per domain; misclassification of duration and intensity is therefore possible. The questionnaire has not been standardised against a criterion measure. Its validity may differ across the age range because parents completed it with younger children, and older children answered independently, and estimates that depend on reported activity volume therefore await confirmation with device-based measurement. Socioeconomic position was operationalised as parental education, a single dimension that does not capture household income, wealth or material circumstances, and income was available only in the junior-high subsample. Residual confounding by unmeasured factors is possible, including pubertal maturation, organised-sport participation, sedentary time, sleep, diet, parental physical activity and access to facilities. This is particularly relevant to the lower fitness of only children, which may reflect unmeasured family or developmental differences. Because the motor battery was administered in the primary grades, the dimension contrasts apply only to those grades and do not extend to grades 7 to 9. The 184 excluded records came disproportionately from children with lower parental education (Supplementary Table S5). Finally, non-significant differences in reported activity are not evidence of equivalence; the confidence intervals bound the size of differences too small to detect but do not rule them out.

The two findings have different implications for monitoring and for programme design. Reporting activity by domain would identify gaps in the activity that families organise, and fitness surveillance that distinguishes strength from trained performance would identify the children with lower performance on particular tests.

## 5. Conclusions

In these schoolchildren, the two family gradients were associated with different outcomes. Socioeconomic position, indexed by parental education, was associated with the amount of activity children reported, and the difference appeared in exercise organised by families; no gradient was observed in the physical education class that every child attends. Family structure was associated with fitness-test performance, with no clear matching difference in reported activity. Body composition was associated with neither family variable. Within this cross-sectional data set, reported activity statistically accounted for little of either family difference in measured fitness. These statistical associations do not estimate what any programme would achieve; they indicate that reported activity alone provides an incomplete description of the relationship between family circumstances and children’s fitness.

## Figures and Tables

**Figure 1:**
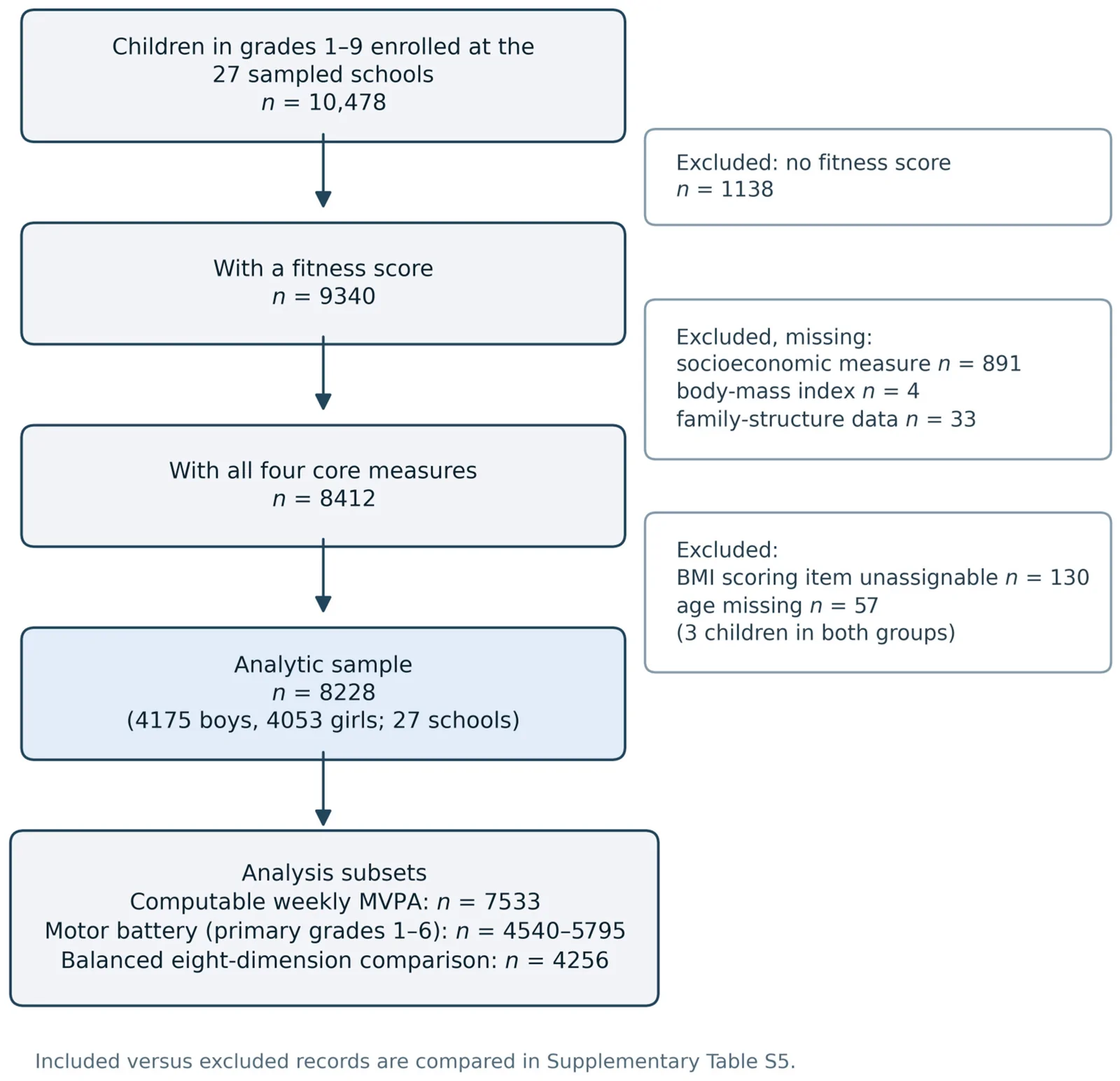
Participant selection. Counts at each selection stage from the enrolled population to the analytic sample, including the reasons for exclusion, the number excluded at each stage, and the sample sizes for the analyses of reported activity, the motor battery and the balanced set of dimensions. Included and excluded records are compared in Supplementary Table S5.

**Figure 2:**
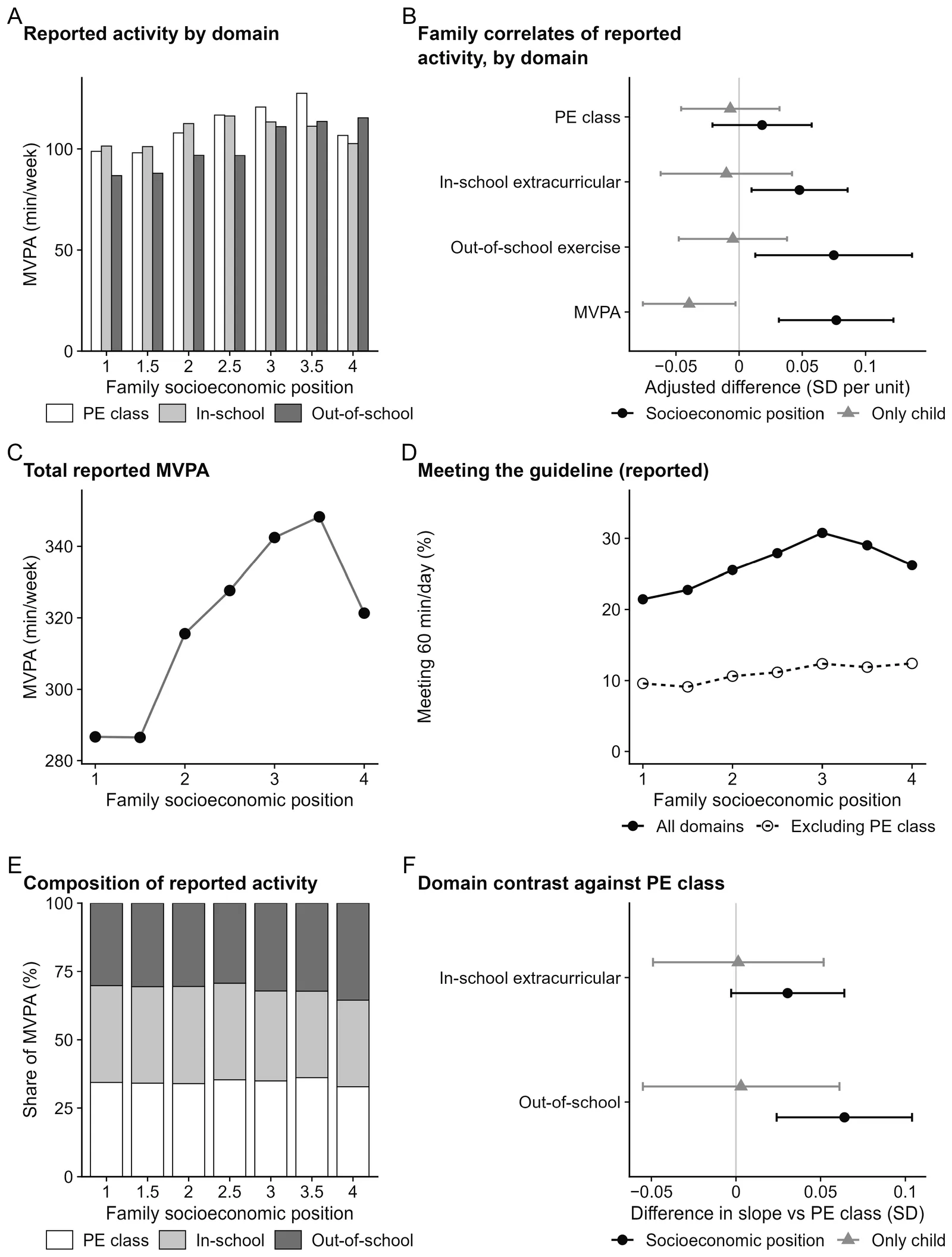
Family gradients in reported physical activity. (**A**) Weekly minutes of moderate-to-vigorous physical activity by domain across levels of family socioeconomic position. (**B**) Adjusted differences in each domain for socioeconomic position and for only-child status. (**C**) Total weekly minutes of moderate-to-vigorous physical activity across levels of socioeconomic position. (**D**) Percentage meeting the guideline of 60 min per day when all domains are included and when the physical education class is excluded. (**E**) Distribution of weekly minutes of moderate-to-vigorous physical activity across domains. (**F**) Difference between the slope for each domain and the slope for the physical education class. All activity quantities are questionnaire-reported.

**Figure 3:**
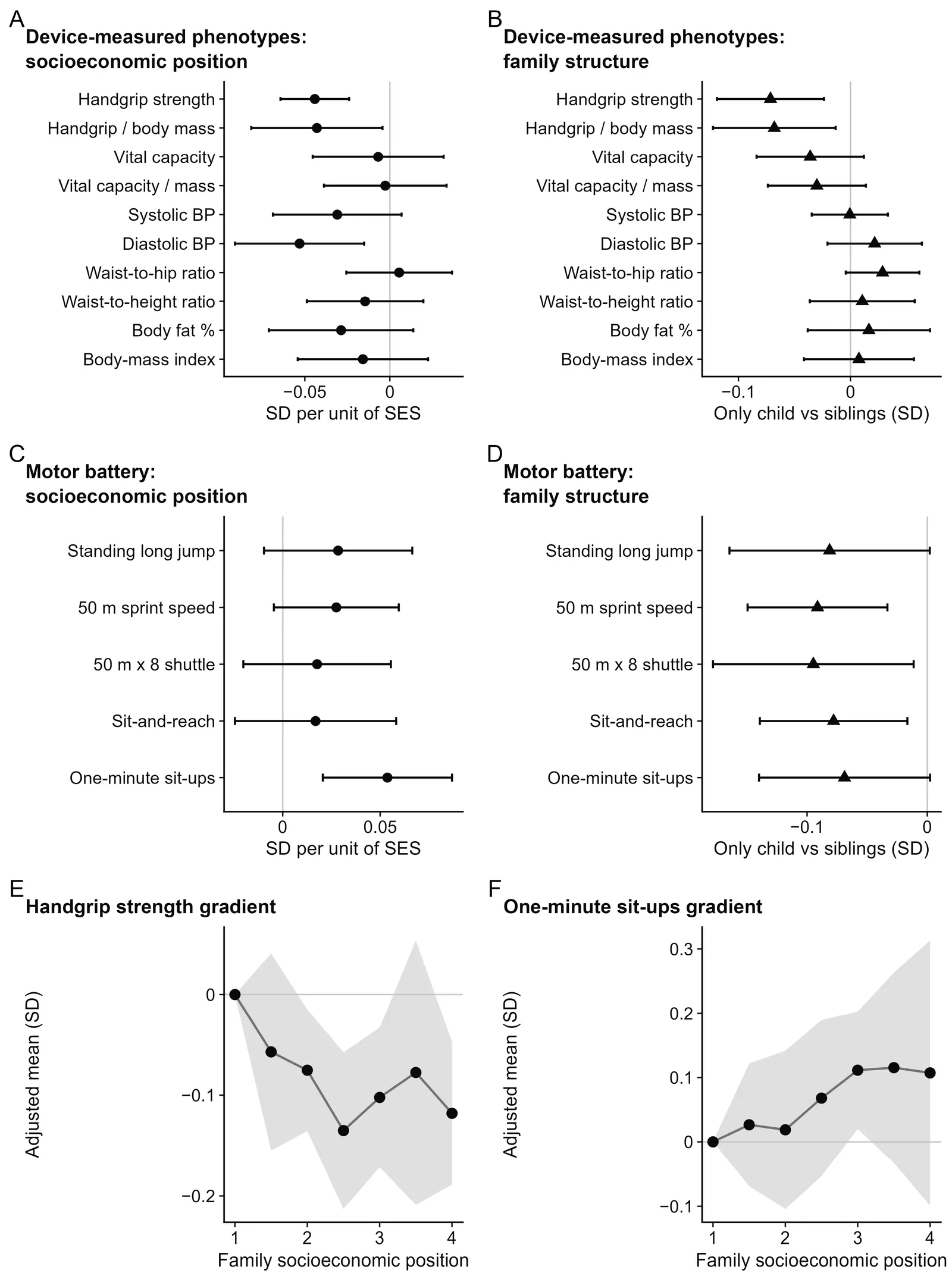
Family circumstances and fitness, by dimension. (**A**) Device-measured phenotypes and socioeconomic position. (**B**) Device-measured phenotypes and only-child status. (**C**) Motor battery and socioeconomic position. (**D**) Motor battery and only-child status. (**E**) Adjusted mean handgrip strength across levels of socioeconomic position. (**F**) Adjusted mean number of one-minute sit-ups across levels of socioeconomic position. Panels (**C**–**F**) are restricted to the primary grades, in which the motor battery was administered.

**Figure 4:**
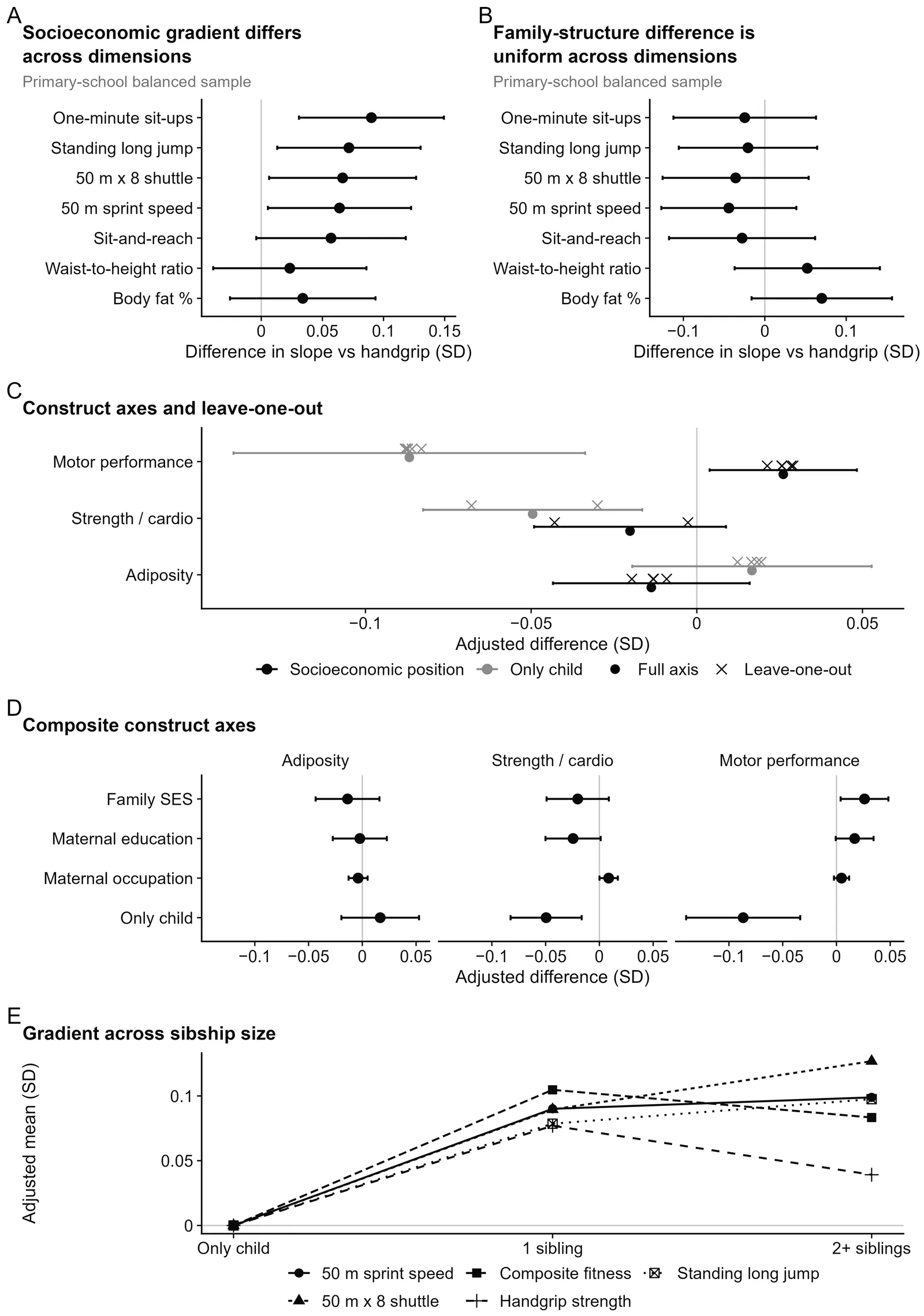
Dimension-specific slopes. (**A**) Socioeconomic slopes relative to the handgrip-strength slope in the balanced primary-school sample. (**B**) Only-child slopes relative to the handgrip-strength slope in the same sample. (**C**) Composite construct axes with leave-one-out estimates. (**D**) Composite construct axes for four family exposures. (**E**) Adjusted means across sibship size. The indicators entering each construct axis are listed in Supplementary Table S8. In (**C**), black denotes socioeconomic position and grey denotes only-child status; filled circles show full-axis estimates and crosses show leave-one-out estimates.

**Figure 5:**
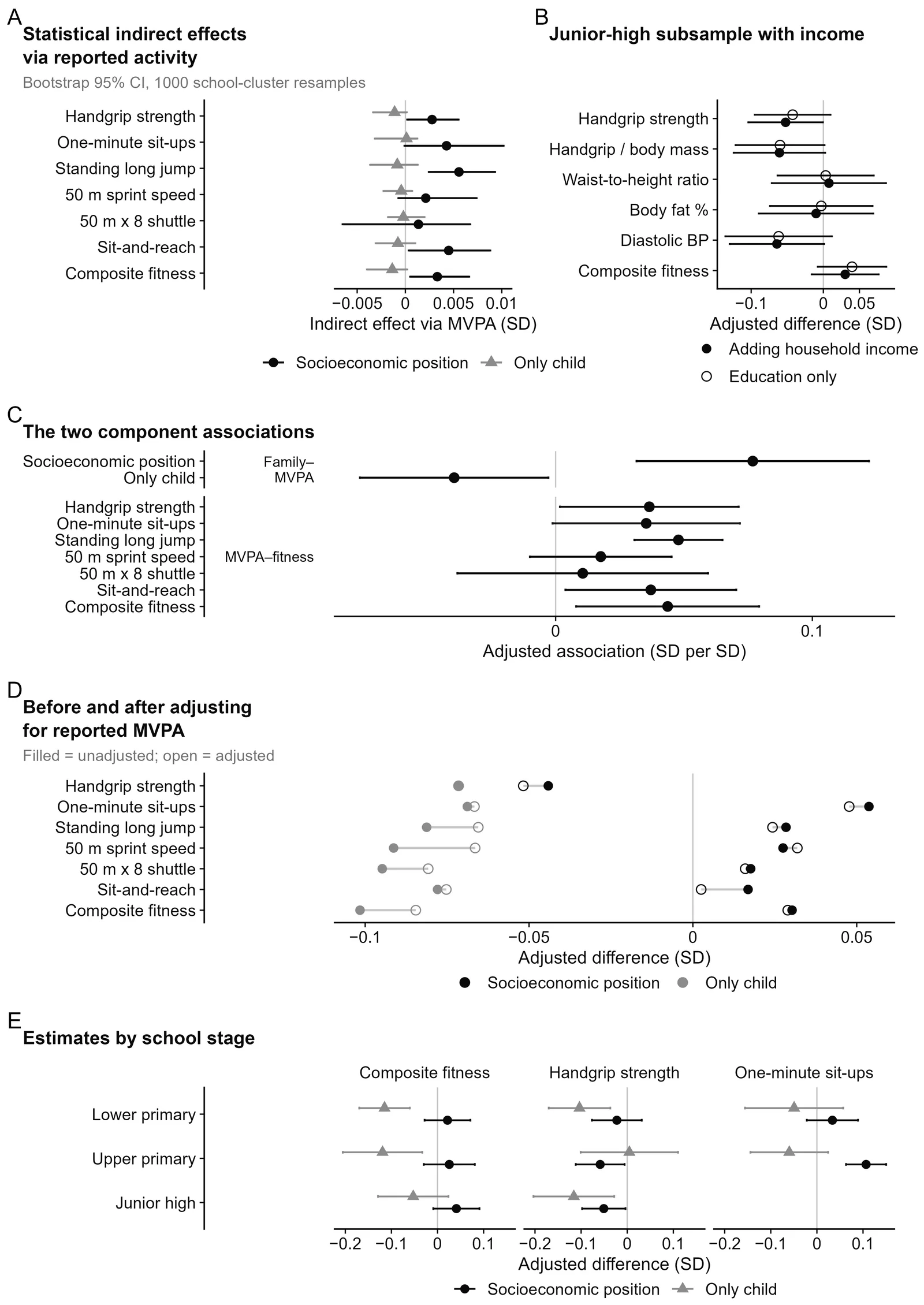
Cross-sectional statistical decomposition through reported activity. (**A**) Statistical indirect effects via weekly moderate-to-vigorous activity with bootstrap intervals. (**B**) Junior-high subsample with reported household income. (**C**) The two component associations: family circumstances with reported activity, and reported activity with fitness. (**D**) Estimates before and after adjustment for weekly reported moderate-to-vigorous activity. (**E**) Estimates by school stage. The decomposition partitions associations in cross-sectional data and implies no temporal or causal ordering. In (**B**), filled circles show estimates with household income added and open circles show estimates with parental education only; in (**D**), filled circles show unadjusted and open circles show adjusted estimates.

**Table 1:** Measurement battery: construct axis, instrument, unit, grades with available data, and distribution by sex.

Measure	Construct Axis	Instrument	Unit	Grades	*n*	Boys Mean (SD)	Girls Mean (SD)
Handgrip strength	Muscular strength	Handgrip dynamometer	kg	1–9	8151	17.71 (9.52)	14.36 (6.53)
Handgrip per body mass	Muscular strength	Derived	kg/kg	1–9	8151	0.40 (0.13)	0.35 (0.10)
Vital capacity	Cardiorespiratory	Electronic spirometer	mL	1–9	8204	1913.79 (986.36)	1576.25 (728.08)
Vital capacity per body mass	Cardiorespiratory	Derived	mL/kg	1–9	8204	43.39 (15.47)	38.57 (13.51)
Systolic blood pressure	Cardiovascular	Sphygmomanometer	mm Hg	1–9	8184	106.34 (14.59)	103.81 (13.41)
Diastolic blood pressure	Cardiovascular	Sphygmomanometer	mm Hg	1–9	8184	69.64 (8.91)	70.42 (8.72)
Waist-to-hip ratio	Body composition	Tape measure, derived	ratio	1–9	8205	0.84 (0.07)	0.81 (0.07)
Waist-to-height ratio	Body composition	Tape measure, derived	ratio	1–9	8205	0.47 (0.06)	0.45 (0.05)
Body fat percentage	Body composition	Bioelectrical impedance	%	1–9	8188	14.28 (6.61)	17.31 (8.12)
Body-mass index	Body composition	Stadiometer and scale, derived	kg/m^2^	1–9	8228	19.51 (4.35)	18.79 (3.89)
Standing long jump	Motor performance	Field test	cm	1–6	5764	133.95 (24.22)	124.25 (20.84)
50 m sprint speed	Motor performance	Field test, timed	m/s	1–6	5745	4.76 (0.62)	4.60 (0.54)
50 m x 8 shuttle speed	Motor performance	Field test, timed	m/s	1–6	5583	3.06 (0.50)	3.00 (0.46)
Sit-and-reach	Motor performance	Sit-and-reach box	cm	1–6	5796	4.40 (6.23)	9.10 (6.67)
One-minute sit-ups	Motor performance	Field test, counted	reps	1–6	4540	24.25 (8.71)	25.57 (9.37)
Composite fitness score	Composite	National standard, renormalised	0–100	1–9	8228	66.75 (12.73)	68.96 (12.32)
Physical education class	Activity domain	Questionnaire, 3 items	min/week	1–9	8066	131.91 (60.78)	132.38 (58.78)
In-school extracurricular activity	Activity domain	Questionnaire, 3 items	min/week	1–9	7881	130.31 (99.50)	128.17 (99.75)
Out-of-school exercise	Activity domain	Questionnaire, 3 items	min/week	1–9	7871	129.07 (116.50)	109.74 (106.55)
Weekly MVPA	Activity summary	Derived from three domains	min/week	1–9	7533	323.93 (221.36)	309.46 (213.03)

**Table 2:** Participant characteristics by sex.

Characteristic	Boys	Girls	*p*
*n*	4175	4053	
Age, years, mean (SD)	10.55 (2.53)	10.64 (2.58)	0.103
Lower primary (grades 1–3), *n* (%)	1772 (42.4)	1659 (40.9)	
Upper primary (grades 4–6), *n* (%)	1227 (29.4)	1161 (28.6)	
Junior high (grades 7–9), *n* (%)	1176 (28.2)	1233 (30.4)	
District 1, *n* (%)	348 (8.3)	292 (7.2)	
District 2, *n* (%)	1115 (26.7)	958 (23.6)	
District 3, *n* (%)	915 (21.9)	918 (22.6)	
District 4, *n* (%)	649 (15.5)	654 (16.1)	
District 5, *n* (%)	1148 (27.5)	1231 (30.4)	
Only child, *n* (%)	1362 (32.6)	930 (22.9)	<0.001
Children in household: 1, *n* (%)	1362 (32.6)	930 (22.9)	
Children in household: 2, *n* (%)	2452 (58.7)	2554 (63.0)	
Children in household: 3 or more, *n* (%)	361 (8.6)	569 (14.0)	
Family socioeconomic position, mean (SD)	2.22 (0.84)	2.15 (0.85)	<0.001
Maternal education, *n* (%)			
Junior high school or below	1213 (29.1)	1299 (32.1)	
Senior or vocational high school	1119 (26.9)	1080 (26.7)	
College or associate degree	1658 (39.8)	1519 (37.6)	
Postgraduate degree	177 (4.2)	144 (3.6)	
Maternal occupation, *n* (%)			
Farming or unemployed	267 (6.4)	238 (5.9)	
Manual	485 (11.7)	450 (11.2)	
Service	190 (4.6)	155 (3.9)	
Clerical	1157 (27.9)	1074 (26.7)	
Technical	170 (4.1)	182 (4.5)	
Professional	193 (4.7)	179 (4.5)	
Managerial	8 (0.2)	4 (0.1)	
Self-employed	1274 (30.7)	1284 (32.0)	
Other	401 (9.7)	452 (11.2)	
Han ethnicity, *n* (%)	3936 (98.5)	3771 (98.0)	

**Table 3:** Associations of family socioeconomic position and family structure with reported weekly activity and fitness outcomes, overall and by sex.

		Family Socioeconomic Position					Only Child				
Outcome	*n*	b (95% CI)	FDR *p*	Boys	Girls	Interaction *p*	b (95% CI)	FDR *p*	Boys	Girls	Interaction *p*
Weekly MVPA	7532	0.077 (0.032 to 0.122)	0.006	0.089	0.067	0.027	−0.040 (−0.076 to −0.003)	0.215	−0.036	−0.040	0.515
Physical education class	8065	0.018 (−0.021 to 0.058)	0.509	0.032	0.004	0.358	−0.007 (−0.046 to 0.032)	0.911	−0.004	−0.011	0.767
In-school extracurricular activity	7880	0.048 (0.010 to 0.086)	0.031	0.069	0.028	0.014	−0.010 (−0.062 to 0.042)	0.911	0.033	−0.068	0.026
Out-of-school exercise	7870	0.075 (0.013 to 0.137)	0.031	0.092	0.060	0.009	−0.005 (−0.048 to 0.038)	0.911	−0.013	0.007	0.704
Handgrip strength	8150	−0.044 (−0.064 to −0.024)	<0.001	−0.012	−0.080	0.794	−0.071 (−0.119 to −0.024)	0.034	−0.052	−0.095	0.326
Handgrip per body mass	8150	−0.043 (−0.082 to −0.004)	0.098	0.004	−0.092	0.995	−0.068 (−0.123 to −0.013)	0.075	−0.056	−0.089	0.724
Vital capacity	8203	−0.007 (−0.045 to 0.032)	0.810	−0.029	0.012	0.278	−0.036 (−0.084 to 0.012)	0.354	−0.001	−0.076	0.127
Vital capacity per body mass	8203	−0.003 (−0.039 to 0.033)	0.884	−0.013	0.006	0.135	−0.030 (−0.074 to 0.014)	0.359	−0.009	−0.059	0.411
Systolic blood pressure	8183	−0.031 (−0.069 to 0.007)	0.273	−0.035	−0.030	0.290	−0.001 (−0.035 to 0.033)	0.975	0.022	−0.025	0.207
Diastolic blood pressure	8183	−0.053 (−0.091 to −0.015)	0.031	−0.081	−0.028	0.627	0.022 (−0.021 to 0.064)	0.525	0.012	0.038	0.649
Waist-to-hip ratio	8204	0.005 (−0.026 to 0.037)	0.810	0.007	0.005	0.082	0.029 (−0.004 to 0.062)	0.291	0.044	0.004	0.178
Waist-to-height ratio	8204	−0.015 (−0.049 to 0.020)	0.597	−0.013	−0.015	0.063	0.011 (−0.036 to 0.057)	0.824	0.039	−0.027	0.056
Body fat percentage	8187	−0.029 (−0.071 to 0.014)	0.372	−0.046	−0.009	0.795	0.016 (−0.038 to 0.071)	0.793	0.025	0.008	0.601
Body-mass index	8227	−0.016 (−0.054 to 0.023)	0.597	−0.020	−0.011	0.055	0.008 (−0.042 to 0.057)	0.848	0.027	−0.012	0.407
Standing long jump	5763	0.028 (−0.010 to 0.066)	0.239	0.022	0.035	0.689	−0.081 (−0.165 to 0.002)	0.058	−0.110	−0.045	0.299
50 m sprint speed	5744	0.028 (−0.005 to 0.060)	0.231	0.004	0.050	0.034	−0.091 (−0.150 to −0.033)	0.011	−0.109	−0.070	0.171
50 m x 8 shuttle speed	5582	0.018 (−0.020 to 0.055)	0.425	−0.015	0.050	0.045	−0.095 (−0.178 to −0.011)	0.043	−0.143	−0.041	0.121
Sit-and-reach	5795	0.017 (−0.025 to 0.058)	0.425	0.002	0.031	0.007	−0.078 (−0.139 to −0.017)	0.032	−0.074	−0.083	0.587
One-minute sit-ups	4540	0.054 (0.021 to 0.087)	0.007	−0.003	0.086	<0.001	−0.069 (−0.140 to 0.002)	0.058	−0.098	−0.031	0.004
Composite fitness score	8227	0.030 (−0.0003 to 0.061)	0.052	0.010	0.047	<0.001	−0.102 (−0.142 to −0.061)	<0.001	−0.125	−0.070	0.032

FDR *p* values are Benjamini–Hochberg-adjusted within the prespecified families defined in Section 2.5 (seven activity outcomes; ten device-measured indicators; five motor-battery items); the composite fitness score is a single prespecified outcome, and its *p* value is unadjusted. Two of the seven activity outcomes, walking time to the usual exercise venue and use of community sports facilities, were included in the multiple-testing correction and are not tabulated; neither met the corrected significance threshold for either exposure. The columns for boys and girls show sex-stratified point estimates; full confidence intervals are provided in Supplementary Table S11. Estimates additionally adjusted for body-mass index are presented as BMI-adjusted sensitivity estimates in Supplementary Table S2. Sex-stratified estimates and interaction *p* values are exploratory (Section 2.5). The lower confidence limit for the socioeconomic-position estimate for the composite fitness score is shown to four decimal places, because it rounds to zero at three decimal places.

## Data Availability

The survey was designed and conducted by the authors. The data are not publicly available because they contain information that could compromise the privacy of the participating children and their families. Enquiries regarding access should be directed to the corresponding author.

## Supplementary Materials

The following supporting information can be downloaded at https://www.mdpi.com/article/10.3390/healthcare14193189/s1, Table S1: STROBE checklist for cross-sectional studies; Table S2: Estimates additionally adjusted for body-mass index; Table S3: Sensitivity analysis for the definition of socioeconomic position; Table S4: Weekly moderate-to-vigorous physical activity by sex and school stage; Table S5: Comparison of included and excluded records; Table S6: Estimates with and without winsorisation; Table S7: Occupation-based socioeconomic indicators; Table S8: Composition of the construct axes; Table S9: Regression diagnostics; Table S10: Restriction to children born after the 2013 policy relaxation; Table S11: Sex-stratified estimates with confidence intervals.
