# Supplementary material for: Two Family Gradients in Children’s Physical Activity and Fitness: Socioeconomic Position, Family Structure, and Where They Diverge
Source: Healthcare (Basel). 2026 Sep 27;14(19):3189. doi: 10.3390/healthcare14193189 (PMC13665455; doi:10.3390/healthcare14193189)
Supplement: Supplementary file 1 [file healthcare-14-03189-s001.zip › healthcare-4531699-supplementary.pdf]

Supplementary Material

Two Family Gradients in Children's Physical Activity and Fitness: Socioeconomic Position, Family Structure, and Where They Diverge

Contents

Supplementary Table S1. STROBE checklist for cross-sectional studies.

Supplementary Table S2. Estimates additionally adjusted for body-mass index.

Supplementary Table S3. Sensitivity analysis for the definition of socioeconomic position.

Supplementary Table S4. Weekly moderate-to-vigorous physical activity by sex and school stage.

Supplementary Table S5. Comparison of included and excluded records.

Supplementary Table S6. Estimates with and without winsorisation.

Supplementary Table S7. Occupation-based socioeconomic indicators.

Supplementary Table S8. Composition of the construct axes.

Supplementary Table S9. Regression diagnostics.

Supplementary Table S10. Restriction to children born after the 2013 policy relaxation.

Supplementary Table S11. Sex-stratified estimates with confidence intervals.

Supplementary Table S1. STROBE checklist for cross-sectional studies.

Item	Recommendation	Reported in
1a	Study design indicated in the title or abstract	Abstract; Methods
1b	Informative and balanced summary in the abstract	Abstract
2	Scientific background and rationale	Introduction
3	Specific objectives, including prespecified questions	Introduction
4	Key elements of study design	Methods 2.1
5	Setting, locations and relevant dates	Methods 2.1
6a	Eligibility criteria, sources and methods of selection	Methods 2.1
7	Outcomes, exposures, predictors and effect modifiers defined	Methods 2.2, 2.3, 2.4
8	Sources of data and details of measurement	Methods 2.2, 2.3
9	Efforts to address potential sources of bias	Methods 2.4, 2.5
10	Study size	Methods 2.1
11	Handling of quantitative variables and groupings	Methods 2.2, 2.3, 2.4
12a	Statistical methods, including confounding control	Methods 2.5
12b	Methods used to examine subgroups and interactions	Methods 2.5
12c	Handling of missing data	Methods 2.2
12e	Sensitivity analyses	Methods 2.5; Supplementary Tables S2, S3
13	Numbers of participants at each stage	Methods 2.1
14a	Characteristics of participants	Table 2

14b	Number of participants with missing data for each variable	Tables 1, 2
15	Outcome data reported	Results 3.1; Table 1
16a	Unadjusted and confounder-adjusted estimates with confidence intervals	Table 3; Supplementary Table S2
16b	Category boundaries reported when continuous variables were categorised	Results 3.3; Figure 3
17	Other analyses, including subgroups and sensitivity analyses	Results 3.4, 3.5; Figures 4, 5; Supplementary Tables S2, S3
18	Key results summarised with reference to study objectives	Discussion
19	Limitations	Discussion
20	Cautious overall interpretation	Discussion
21	Generalisability	Discussion
22	Source of funding and role of the funders	Title page

Supplementary Table S2. Estimates additionally adjusted for body-mass index.

Outcome	Exposure	Adjusted for BMI, b (95% CI)	P	n
Handgrip strength	Socioeconomic position	-0.040 (-0.063 to -0.017)	0.001	8150
Handgrip strength	Only child	-0.080 (-0.130 to -0.031)	0.001	8150
Handgrip per body mass	Socioeconomic position	-0.050 (-0.077 to -0.023)	0.000	8150
Handgrip per body mass	Only child	-0.052 (-0.096 to -0.007)	0.023	8150
Vital capacity	Socioeconomic position	-0.003 (-0.039 to 0.033)	0.850	8203
Vital capacity	Only child	-0.044 (-0.087 to 0.000)	0.051	8203
Vital capacity per body mass	Socioeconomic position	-0.010 (-0.045 to 0.026)	0.592	8203
Vital capacity per body mass	Only child	-0.014 (-0.061 to 0.033)	0.552	8203
Systolic blood pressure	Socioeconomic position	-0.025 (-0.060 to 0.009)	0.144	8183
Systolic blood pressure	Only child	-0.013 (-0.047 to 0.021)	0.458	8183
Diastolic blood pressure	Socioeconomic position	-0.051 (-0.089 to -0.012)	0.011	8183
Diastolic blood pressure	Only child	0.016 (-0.027 to 0.059)	0.465	8183
Standing long jump	Socioeconomic position	0.022 (-0.014 to 0.059)	0.236	5763
Standing long jump	Only child	-0.067 (-0.144 to 0.009)	0.086	5763
50-m sprint speed	Socioeconomic position	0.023 (-0.007 to 0.053)	0.140	5744
50-m sprint speed	Only child	-0.078 (-0.133 to -0.024)	0.005	5744
50-m x 8 shuttle speed	Socioeconomic position	0.014 (-0.019 to 0.047)	0.414	5582
50-m x 8 shuttle speed	Only child	-0.081 (-0.155 to -0.007)	0.031	5582
Sit-and-reach	Socioeconomic position	0.017 (-0.025 to 0.059)	0.427	5795
Sit-and-reach	Only child	-0.078 (-0.141 to -0.016)	0.014	5795
One-minute sit-ups	Socioeconomic position	0.054 (0.023 to 0.084)	0.001	4540
One-minute sit-ups	Only child	-0.066 (-0.133 to 0.001)	0.052	4540
Composite fitness score	Socioeconomic position	0.028 (-0.005 to 0.060)	0.092	8227

Composite fitness score	Only child	-0.096 (-0.137 to -0.054)	0.000	8227

Supplementary Table S3. Sensitivity analysis for the definition of socioeconomic position.

Outcome	Main: mean parental education	Sensitivity: highest household education	Sensitivity: additionally adjusted for BMI	n
Weekly MVPA	0.077 (0.032 to 0.122)	0.063 (0.023 to 0.103)		7532
Physical education class	0.018 (-0.021 to 0.058)	0.023 (-0.011 to 0.056)		8065
In-school extracurricular activity	0.048 (0.010 to 0.086)	0.044 (0.014 to 0.074)		7880
Out-of-school exercise	0.075 (0.013 to 0.137)	0.058 (0.004 to 0.111)		7870
Handgrip strength	-0.044 (-0.064 to -0.024)	-0.045 (-0.067 to -0.024)	-0.040 (-0.063 to -0.017)	8150
Handgrip per body mass	-0.043 (-0.082 to -0.004)	-0.044 (-0.082 to -0.007)	-0.050 (-0.077 to -0.023)	8150
Vital capacity per body mass	-0.003 (-0.039 to 0.033)	-0.013 (-0.043 to 0.017)	-0.010 (-0.045 to 0.026)	8203
Systolic blood pressure	-0.031 (-0.069 to 0.007)	-0.031 (-0.061 to -0.001)		8183
Diastolic blood pressure	-0.053 (-0.091 to -0.015)	-0.055 (-0.091 to -0.018)		8183
Waist-to-hip ratio	0.005 (-0.026 to 0.037)	0.007 (-0.022 to 0.036)		8204
Waist-to-height ratio	-0.015 (-0.049 to 0.020)	-0.004 (-0.031 to 0.024)		8204
Body fat percentage	-0.029 (-0.071 to 0.014)	-0.023 (-0.053 to 0.007)		8187
Body-mass index	-0.016 (-0.054 to 0.023)	-0.011 (-0.042 to 0.020)		8227
Standing long jump	0.028 (-0.010 to 0.066)	0.021 (-0.015 to 0.056)	0.022 (-0.014 to 0.059)	5763
50-m sprint speed	0.028 (-0.005 to 0.060)	0.021 (-0.013 to 0.054)	0.023 (-0.007 to 0.053)	5744
50-m x 8 shuttle speed	0.018 (-0.020 to 0.055)	0.022 (-0.012 to 0.055)	0.014 (-0.019 to 0.047)	5582
Sit-and-reach	0.017 (-0.025 to 0.058)	0.014 (-0.032 to 0.059)	0.017 (-0.025 to 0.059)	5795
One-minute sit-ups	0.054 (0.021 to 0.087)	0.047 (0.014 to 0.079)	0.054 (0.023 to 0.084)	4540
Composite fitness score	0.030 (-0.0003 to 0.061)	0.016 (-0.013 to 0.045)	0.028 (-0.005 to 0.060)	8227

Supplementary Table S4. Weekly moderate-to-vigorous physical activity by sex and school stage.

Group	n	Mean (SD), min/wk	Median (P25–P75), min/wk	Meeting 60 min/day, %

All children	7,533	316.8 (217.4)	300 (165–450)	26.3
Boys	3,805	323.9 (221.4)	315 (180–450)	28.0
Girls	3,728	309.5 (213.0)	285 (150–405)	24.6
Lower primary (grades 1–3)	3,056	334.2 (223.7)	315 (180–450)	30.5
Upper primary (grades 4–6)	2,251	315.7 (215.0)	315 (150–450)	29.1
Junior high (grades 7–9)	2,226	293.9 (208.7)	270 (150–405)	17.8

MVPA, moderate-to-vigorous physical activity. n = children with computable weekly MVPA (7,533 of 8,228). The boys–girls difference, adjusted for age and school fixed effects with school-clustered standard errors, was 15.1 min/wk (95% CI 4.2 to 26.0).

Supplementary Table S5. Comparison of included (n = 8,228) and excluded (n = 184) records among the 8,412 children with the four core measures.

**Characteristic**	**Included**	**Excluded**	**P**
Age, years	10.59 (2.56)	10.47 (2.49)	.588
Girls, n (%)	4053 (49.3%)	85 (46.2%)	.455
Socioeconomic position (parental education, 1-4)	2.19 (0.84)	1.89 (0.84)	<.001
Only child, n (%)	2292 (27.9%)	46 (25.0%)	.440
Number of children in household	1.83 (0.60)	1.89 (0.62)	.218
Body-mass index, kg/m2	19.15 (4.15)	19.75 (4.01)	.048
Composite fitness score (BMI item removed)	67.84 (12.57)	62.53 (11.59)	.002
Lower primary (grades 1-3), n (%)	3431 (41.7%)	61 (33.2%)	.024
Upper primary (grades 4-6), n (%)	2388 (29.0%)	32 (17.4%)	<.001
Junior high (grades 7-9), n (%)	2409 (29.3%)	91 (49.5%)	<.001

Continuous variables: mean (SD), Welch t-test; categorical: n (%), chi-squared test. Among excluded records, age was available for 127 and the composite fitness score for 54; percentages use available records. Exclusion reasons: body-mass-index scoring item unassignable (130), age missing (57), 3 records lacking both.

Supplementary Table S6. Family gradients in reported activity with and without winsorisation of weekly totals at the 99.5th percentile.

Exposure	Outcome	Winsorised b (95% CI)	P	Unwinsorised b (95% CI)	P	n
Socioeconomic position	Total reported activity	0.069 (0.019 to 0.119)	.007	0.067 (0.018 to 0.115)	.007	7645
Socioeconomic position	Weekly MVPA	0.077 (0.032 to 0.122)	<.001	0.074 (0.030 to 0.119)	.001	7532
Socioeconomic position	Physical education class	0.018 (-0.021 to 0.058)	.364	0.020 (-0.021 to 0.060)	.340	8065
Socioeconomic position	In-school extracurricular activity	0.048 (0.010 to 0.086)	.014	0.046 (0.010 to 0.082)	.012	7880
Socioeconomic position	Out-of-school exercise	0.075 (0.013 to 0.137)	.018	0.075 (0.013 to 0.137)	.018	7870
Only child	Total reported activity	-0.002 (-0.043 to 0.039)	.911	-0.004 (-0.046 to 0.038)	.864	7645
Only child	Weekly MVPA	-0.040 (-0.076 to -0.003)	.035	-0.039 (-0.075 to -0.002)	.036	7532
Only child	Physical education class	-0.007 (-0.046 to 0.032)	.727	-0.010 (-0.050 to 0.029)	.615	8065
Only child	In-school extracurricular activity	-0.010 (-0.062 to 0.042)	.704	-0.006 (-0.061 to 0.050)	.842	7880
Only child	Out-of-school exercise	-0.005 (-0.048 to 0.038)	.822	-0.005 (-0.048 to 0.038)	.822	7870

Winsorisation capped 36 children at the 99.5th percentile of weekly totals. The guideline indicator is unaffected because the cap lies far above 420 min/week. Models as in the main analysis (SD units; school fixed effects; school-clustered standard errors).

Supplementary Table S7. Occupation-based socioeconomic indicators and the main outcome families.

Exposure	Outcome	b or OR (95% CI)	P	FDR P	n
Maternal occupational prestige	Total activity	0.018 (-0.013 to 0.050)	.257	.426	5218
Maternal occupational prestige	Weekly MVPA	0.020 (-0.011 to 0.050)	.203	.426	5146
Maternal occupational prestige	PE class	-0.014 (-0.040 to 0.012)	.304	.426	5487

Maternal occupational prestige	In-school extracurricular	0.022 (-0.002 to 0.046)	.077	.426	5370
Maternal occupational prestige	Out-of-school exercise	0.018 (-0.015 to 0.052)	.278	.426	5369
Maternal occupational prestige	Walking time to venue	0.000 (-0.022 to 0.022)	.998	.998	5360
Maternal occupational prestige	Community facility use	-0.011 (-0.052 to 0.030)	.602	.702	5179
Maternal occupational prestige	Handgrip strength	-0.002 (-0.024 to 0.020)	.861	.952	5553
Maternal occupational prestige	Handgrip per body mass	-0.020 (-0.038 to -0.002)	.026	.264	5553
Maternal occupational prestige	Vital capacity	-0.013 (-0.039 to 0.014)	.348	.753	5588
Maternal occupational prestige	Vital capacity per body mass	-0.019 (-0.055 to 0.017)	.312	.753	5588
Maternal occupational prestige	Systolic blood pressure	-0.003 (-0.029 to 0.024)	.847	.952	5573
Maternal occupational prestige	Diastolic blood pressure	-0.014 (-0.043 to 0.015)	.340	.753	5573
Maternal occupational prestige	Waist-to-hip ratio	0.013 (-0.016 to 0.041)	.376	.753	5583
Maternal occupational prestige	Waist-to-height ratio	-0.001 (-0.034 to 0.032)	.952	.952	5583
Maternal occupational prestige	Body fat percentage	0.015 (-0.031 to 0.061)	.520	.867	5575
Maternal occupational prestige	Body-mass index	0.008 (-0.026 to 0.041)	.658	.940	5604
Maternal occupational prestige	Standing long jump	-0.007 (-0.031 to 0.016)	.526	.866	3994
Maternal occupational prestige	50-m sprint speed	-0.002 (-0.032 to 0.027)	.866	.866	3978
Maternal occupational prestige	50-m x 8 shuttle speed	0.013 (-0.030 to 0.056)	.559	.866	3865
Maternal occupational	Sit-and-reach	-0.003 (-0.031 to	.856	.866	4017

prestige		0.026)			
Maternal occupational prestige	One-minute sit-ups	0.025 (-0.003 to 0.052)	.076	.378	3140
Maternal occupational prestige	Composite fitness score	0.005 (-0.018 to 0.029)	.657	.657	5604
Maternal occupational prestige	Meeting guideline (OR)	1.05 (0.97 to 1.13)	.257	-	5147
Paternal occupational prestige	Total activity	0.023 (-0.002 to 0.049)	.076	.265	5365
Paternal occupational prestige	Weekly MVPA	0.010 (-0.019 to 0.039)	.498	.697	5285
Paternal occupational prestige	PE class	0.022 (-0.026 to 0.070)	.365	.639	5662
Paternal occupational prestige	In-school extracurricular	0.021 (0.003 to 0.039)	.021	.146	5530
Paternal occupational prestige	Out-of-school exercise	0.005 (-0.027 to 0.038)	.755	.881	5527
Paternal occupational prestige	Walking time to venue	0.018 (-0.015 to 0.052)	.275	.639	5535
Paternal occupational prestige	Community facility use	0.002 (-0.050 to 0.054)	.949	.949	5342
Paternal occupational prestige	Handgrip strength	-0.010 (-0.038 to 0.018)	.485	.809	5718
Paternal occupational prestige	Handgrip per body mass	-0.018 (-0.052 to 0.016)	.290	.808	5718
Paternal occupational prestige	Vital capacity	-0.013 (-0.042 to 0.015)	.366	.808	5762
Paternal occupational prestige	Vital capacity per body mass	-0.018 (-0.050 to 0.013)	.243	.808	5762
Paternal occupational prestige	Systolic blood pressure	-0.023 (-0.064 to 0.017)	.257	.808	5748
Paternal occupational prestige	Diastolic blood pressure	0.006 (-0.023 to 0.034)	.702	.878	5748
Paternal occupational prestige	Waist-to-hip ratio	-0.007 (-0.034 to 0.020)	.612	.874	5757

Paternal occupational prestige	Waist-to-height ratio	-0.017 (-0.056 to 0.023)	.404	.808	5757
Paternal occupational prestige	Body fat percentage	-0.005 (-0.052 to 0.042)	.828	.920	5748
Paternal occupational prestige	Body-mass index	-0.0004 (-0.047 to 0.046)	.988	.988	5777
Paternal occupational prestige	Standing long jump	0.012 (-0.024 to 0.048)	.504	.630	4148
Paternal occupational prestige	50-m sprint speed	0.022 (-0.002 to 0.047)	.074	.370	4130
Paternal occupational prestige	50-m x 8 shuttle speed	0.015 (-0.027 to 0.057)	.489	.630	4023
Paternal occupational prestige	Sit-and-reach	0.014 (-0.007 to 0.035)	.194	.486	4171
Paternal occupational prestige	One-minute sit-ups	0.005 (-0.038 to 0.048)	.825	.825	3250
Paternal occupational prestige	Composite fitness score	0.013 (-0.013 to 0.039)	.326	.326	5777
Paternal occupational prestige	Meeting guideline (OR)	0.99 (0.91 to 1.08)	.826	-	5286

Occupation was recorded in nine categories and recoded to a four-level prestige score (managerial and professional = 4; clerical = 3; commerce, service and military = 2.5; production and transport = 2; agriculture = 1.5; not employed = 1; other = missing), following the convention used for this survey. Models adjust for only-child status, age and school fixed effects with school-clustered standard errors; FDR correction within each outcome family per exposure. The final row per exposure is the odds ratio for meeting the guideline.

Supplementary Table S8. Composition of the construct axes and the composite fitness score.

**Axis or score**	**Indicators entering**	**Construction**
Motor performance axis	Standing long jump; 50-m sprint speed; 50-m x 8 shuttle speed; sit-and-reach; one-minute sit-ups	Mean of the sex- and age-standardised indicators; higher = better performance
Strength/cardio axis	Handgrip per kilogram of body mass; vital capacity per kilogram of body mass	Mean of the standardised indicators; higher = stronger/larger capacity
Adiposity axis	Waist-to-hip ratio; waist-to-height ratio; body fat percentage; body-mass index	Mean of the standardised indicators; higher = more adipose
Composite fitness score	2014 National Student Physical Health Standard	National scoring weights; higher = better

item scores with the body-mass-index item removed and the remaining weights renormalised (0-100)

Axes are computed as the mean of the available standardised components for each child and re-estimated leaving each component out in turn (Figure 4C).

Supplementary Table S9. Regression diagnostics for the principal models.

Outcome model	VIF (SES)	VIF (only child)	VIF (age)	Residual skewness	Residual kurtosis	Breusch-Pagan chi2	BP P	n
Weekly MVPA	1.16	1.02	1.13	0.78	5.10	20.3	<.001	7532
Handgrip strength	1.16	1.02	1.13	0.33	3.51	1.0	.794	8150
Composite fitness score	1.16	1.02	1.14	-0.62	3.52	8.7	.034	8227
One-minute sit-ups	1.07	1.03	1.04	-0.19	3.17	17.3	<.001	4540

Variance-inflation factors are all at or below 1.2, indicating no problematic collinearity among the exposures and age. Breusch-Pagan tests indicate heteroscedasticity for some outcomes; all reported inference therefore uses school-clustered heteroscedasticity-robust standard errors, which do not assume homoscedastic or normally distributed residuals.

Supplementary Table S10. Only-child estimates in the full sample and restricted to children born after the 2013 relaxation of the one-child policy (age 9 years or younger in 2023).

Outcome	Full sample b (95% CI)	P	n	Post-2013 cohort b (95% CI)	P	n
Composite fitness score	-0.102 (-0.142 to -0.061)	<.001	8227	-0.100 (-0.150 to -0.051)	<.001	3232
Handgrip strength	-0.071 (-0.119 to -0.024)	.003	8150	-0.121 (-0.189 to -0.053)	<.001	3210
Handgrip per body mass	-0.068 (-0.123 to -0.013)	.015	8150	-0.077 (-0.180 to 0.025)	.139	3210
Standing long jump	-0.081 (-0.165 to 0.002)	.056	5763	-0.100 (-0.186 to -0.013)	.024	3198
50-m sprint speed	-0.091 (-0.150 to -0.033)	.002	5744	-0.096 (-0.155 to -0.038)	.001	3185
50-m x 8 shuttle speed	-0.095 (-0.178 to -0.011)	.026	5582	-0.080 (-0.202 to 0.042)	.196	3094

Sit-and-reach	-0.078 (-0.139 to -0.017)	.013	5795	-0.055 (-0.109 to -0.001)	.045	3217
One-minute sit-ups	-0.069 (-0.140 to 0.002)	.058	4540	-0.054 (-0.159 to 0.051)	.309	3120

Models adjust for socioeconomic position, age and school fixed effects with school-clustered standard errors; unadjusted P values shown. The restriction addresses cohort composition under changing fertility policy, not family-level selection within cohorts.

Supplementary Table S11. Sex-stratified estimates with 95% confidence intervals for every outcome in Table 3.

Exposure	Outcome	Boys b (95% CI)	Girls b (95% CI)	Sex-interaction P
Socioeconomic position	Weekly MVPA	0.089 (0.043 to 0.134)	0.067 (0.013 to 0.121)	.027
Socioeconomic position	Physical education class	0.032 (-0.004 to 0.068)	0.004 (-0.050 to 0.059)	.358
Socioeconomic position	In-school extracurricular activity	0.069 (0.021 to 0.117)	0.028 (-0.015 to 0.070)	.014
Socioeconomic position	Out-of-school exercise	0.092 (0.042 to 0.142)	0.060 (-0.026 to 0.146)	.009
Socioeconomic position	Handgrip strength	-0.012 (-0.053 to 0.028)	-0.080 (-0.120 to -0.040)	.794
Socioeconomic position	Handgrip per body mass	0.004 (-0.049 to 0.057)	-0.092 (-0.146 to -0.038)	.995
Socioeconomic position	Vital capacity	-0.029 (-0.076 to 0.019)	0.012 (-0.045 to 0.069)	.278
Socioeconomic position	Vital capacity per body mass	-0.013 (-0.055 to 0.029)	0.006 (-0.059 to 0.070)	.135
Socioeconomic position	Systolic blood pressure	-0.035 (-0.081 to 0.011)	-0.030 (-0.077 to 0.017)	.290
Socioeconomic position	Diastolic blood pressure	-0.081 (-0.128 to -0.034)	-0.028 (-0.080 to 0.023)	.627
Socioeconomic position	Waist-to-hip ratio	0.007 (-0.032 to 0.046)	0.005 (-0.041 to 0.051)	.082
Socioeconomic position	Waist-to-height ratio	-0.013 (-0.050 to 0.023)	-0.015 (-0.072 to 0.042)	.063
Socioeconomic position	Body fat percentage	-0.046 (-0.089 to -0.004)	-0.009 (-0.082 to 0.064)	.795
Socioeconomic position	Body-mass index	-0.020 (-0.054 to 0.014)	-0.011 (-0.073 to 0.051)	.055
Socioeconomic position	Standing long jump	0.022 (-0.037 to 0.080)	0.035 (-0.017 to 0.087)	.689
Socioeconomic position	50-m sprint speed	0.004 (-0.034 to 0.042)	0.050 (-0.001 to 0.101)	.034
Socioeconomic position	50-m x 8 shuttle speed	-0.015 (-0.039 to 0.010)	0.050 (-0.017 to 0.118)	.045
Socioeconomic position	Sit-and-reach	0.002 (-0.055 to 0.059)	0.031 (-0.012 to 0.074)	.007
Socioeconomic position	One-minute sit-ups	-0.003 (-0.091 to 0.085)	0.086 (0.048 to 0.124)	<.001
Socioeconomic position	Composite fitness score	0.010 (-0.033 to 0.053)	0.047 (0.010 to 0.084)	<.001
Only child	Weekly MVPA	-0.036 (-0.082 to 0.010)	-0.040 (-0.097 to 0.017)	.515
Only child	Physical education class	-0.004 (-0.040 to 0.031)	-0.011 (-0.078 to 0.055)	.767
Only child	In-school extracurricular	0.033 (-0.039 to 0.106)	-0.068 (-0.141 to 0.004)	.026

	activity			
Only child	Out-of-school exercise	-0.013 (-0.065 to 0.038)	0.007 (-0.046 to 0.059)	.704
Only child	Handgrip strength	-0.052 (-0.102 to -0.002)	-0.095 (-0.155 to -0.035)	.326
Only child	Handgrip per body mass	-0.056 (-0.111 to -0.0004)	-0.089 (-0.172 to -0.006)	.724
Only child	Vital capacity	-0.001 (-0.063 to 0.061)	-0.076 (-0.146 to -0.006)	.127
Only child	Vital capacity per body mass	-0.009 (-0.062 to 0.044)	-0.059 (-0.132 to 0.014)	.411
Only child	Systolic blood pressure	0.022 (-0.015 to 0.059)	-0.025 (-0.100 to 0.051)	.207
Only child	Diastolic blood pressure	0.012 (-0.051 to 0.075)	0.038 (-0.042 to 0.118)	.649
Only child	Waist-to-hip ratio	0.044 (-0.014 to 0.102)	0.004 (-0.054 to 0.063)	.178
Only child	Waist-to-height ratio	0.039 (-0.025 to 0.103)	-0.027 (-0.104 to 0.050)	.056
Only child	Body fat percentage	0.025 (-0.040 to 0.090)	0.008 (-0.094 to 0.110)	.601
Only child	Body-mass index	0.027 (-0.033 to 0.088)	-0.012 (-0.117 to 0.092)	.407
Only child	Standing long jump	-0.110 (-0.186 to -0.035)	-0.045 (-0.181 to 0.091)	.299
Only child	50-m sprint speed	-0.109 (-0.186 to -0.031)	-0.070 (-0.137 to -0.004)	.171
Only child	50-m x 8 shuttle speed	-0.143 (-0.216 to -0.070)	-0.041 (-0.185 to 0.103)	.121
Only child	Sit-and-reach	-0.074 (-0.134 to -0.013)	-0.083 (-0.186 to 0.020)	.587
Only child	One-minute sit-ups	-0.098 (-0.164 to -0.033)	-0.031 (-0.123 to 0.062)	.004
Only child	Composite fitness score	-0.125 (-0.185 to -0.066)	-0.070 (-0.135 to -0.006)	.032

Estimates in SD units from sex-stratified models with the same covariates as the main analysis; interaction P from a multiplicative exposure-by-sex term in the pooled model. Interaction terms were computed for every outcome and are interpreted as exploratory.
